# Friends and Foes: The Ambivalent Role of Autophagy in HIV-1 Infection

**DOI:** 10.3390/v16040500

**Published:** 2024-03-25

**Authors:** Susanne Klute, Konstantin M. J. Sparrer

**Affiliations:** Institute of Molecular Virology, Ulm University Medical Center, 89081 Ulm, Germany

**Keywords:** HIV, autophagy, innate immunity

## Abstract

Autophagy has emerged as an integral part of the antiviral innate immune defenses, targeting viruses or their components for lysosomal degradation. Thus, successful viruses, like pandemic human immunodeficiency virus 1 (HIV-1), evolved strategies to counteract or even exploit autophagy for efficient replication. Here, we provide an overview of the intricate interplay between autophagy and HIV-1. We discuss the impact of autophagy on HIV-1 replication and report in detail how HIV-1 manipulates autophagy in infected cells and beyond. We also highlight tissue and cell-type specifics in the interplay between autophagy and HIV-1. In addition, we weigh exogenous modulation of autophagy as a putative double-edged sword against HIV-1 and discuss potential implications for future antiretroviral therapy and curative approaches. Taken together, we consider both antiviral and proviral roles of autophagy to illustrate the ambivalent role of autophagy in HIV-1 pathogenesis and therapy.

## 1. Introduction

### 1.1. Regulation of Autophagy

**Figure 1:**
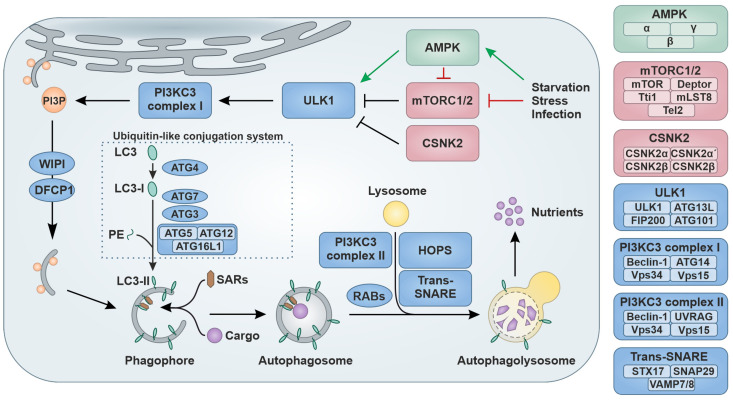
Schematic overview of the autophagy pathway. (**Left panel**) Upon autophagy induction AMPK activates ULK1. Negative regulation is provided by the mTORC1/2 and Casein kinase II complexes. The activation of the PI3KC3 complex I promotes PI3P production at the endoplasmic reticulum. PI3P assembles WIPI proteins and DFCP1 to promote initial phagophore formation. To the phagophore, cytoplasmic cargo is recruited via selective autophagy receptors (SARs). LC3 is proteolytically cleaved by ATG4 to form LC3-I. A ubiquitin-like conjugation process mediated by ATG 7, ATG3 and ATG5-ATG12-ATG16L1 attaches phosphatidylethanolamine (PE) to LC3-I to generate LC3-II, which decorates the inner and outer membrane the phagophore. The phagophore matures into the double-membrane vesicle termed the autophagosome. Mediated by RAB proteins, SNARE proteins, the PI3KC3 complex II and the HOPS complex, the autophagosome fuses with a lysosome, forming the autophagolysosome and leading to the degradation of the cargo, inner membrane and SARs. Green arrows indicate positive stimulation; red arrows indicate negative regulation. (**Right panel**) Core proteins of the complexes involved in autophagy.

Autophagy (coined from Greek: ‘auto’-self and ‘phagein’ eating) is an evolutionary highly conserved homeostatic and intricately regulated cytoplasmic catabolic pathway [1,2,3]. There are three modes of autophagy: macroautophagy, microautophagy and chaperone-mediated autophagy. The most common mode, macroautophagy (hereafter called autophagy), is characterized by the formation of cytosolic double-membrane vesicles (autophagic vesicle = autophagosome) that engulf cargo in the cytoplasm [4]. While originally discovered as a nonselective bulk degradation pathway, it is now established that autophagy can also target cargoes in a highly specific manner (‘selective autophagy’) via dedicated autophagy receptors, such as Sequestosome-1 (SQSTM1/p62) that recruit cargo earmarked, e.g., by ubiquitin [2]. As a stress response, autophagy is activated upon extra- or intracellular stress, such as starvation, elevated temperature, osmotic pressure or pathogen infection (Figure 1) [2,5]. A dedicated set of kinases controls the level of autophagic flux, i.e., the turnover rate of autophagy. For example, activation of the stress sensor 5′ AMP-activated protein kinase (AMPK) comprised of α-, β- and γ-subunits leads to the phosphorylation and activation of the Unc-like kinase 1 complex (ULK1) consisting of ULK1, Autophagy related (ATG) 13L, FAK family kinase-interacting protein of 200 kDa (FIP200) and ATG101 [6]. Negative regulation is provided by two mechanistic targets of rapamycin (mTOR) complexes (mTORC1 and mTORC2) or the heterotetrameric Casein kinase II (CSNK2) complex (CSNK2α, CSNK2α′ and two CSNK2β subunits) that inhibit the activity of the ULK1 complex [7,8]. The active ULK1 complex in turn stimulates the Class III phosphatidylinositol 3-kinase (PI3KC3) complex I, comprised of Beclin-1, ATG14, the kinase Vacuolar protein sorting (Vps) 34 and membrane-anchoring Vps15 [9]. The activity of this complex results in a locally elevated production of Phosphatidylinositol 3-phosphate (PI3P), which in turn recruits WD-repeat protein interacting with phosphoinositides (WIPI) proteins and Double FYVE-containing protein 1 (DFCP1), which is required especially for selective autophagy [10,11]. Next, a double-layer membrane, the so-called phagophore, is assembled in an ATG9-dependent manner with lipids mainly derived from the endoplasmic reticulum (ER) [12,13]. ATG8 proteins like GABA type A receptor-associated proteins (GABARAP) or Microtubule-associated protein 1 light chain 3 (MAP1BLC3) isoforms (short LC3A, LC3B, and LC3C) undergo ubiquitin-like modification during phagophore elongation [14]. For example, LC3 proteins are proteolytically processed by ATG4 to generate LC3-I. Then, in a ubiquitin-like conjugation process, Phosphatidylethanolamine (PE) is covalently attached to LC3-I forming LC3-II. Here, ATG7 serves as an E1-like enzyme, ATG3 acts as the E2-like enzyme and the ATG5–ATG12–ATG16L1 complex represents the E3-ligase-like protein ligase [15,16]. LC3-II is then inserted into the phagophore and earmarked cargo is recruited via selective autophagy receptors (SARs) such as Neighbor of BRCA1 gene 1 (NBR1), Optineurin (OPTN) or SQSTM1/p62 [17,18]. In precision autophagy, target recognition is achieved independent of protein tags and directly recognized and recruited by specific receptors, e.g., Tripartite motif (TRIM) proteins [19,20,21,22]. During maturation, the phagophore closes to form a double-membrane vesicle called the autophagosome. The transition of cytoplasmic LC3 (LC3-I) into its PE-conjugated version (LC3-II), which decorates phagophores and autophagosomes, is a hallmark of autophagy [23]. Subsequently, fusion of the autophagosome with a lysosome to form the autophagolysosome is promoted by the PI3KC3 complex II, consisting of Beclin-1, UV radiation resistance-associated gene protein (UVRAG), Vps34 and Vps15, several small Ras-associated binding (RAB) GTPases, Soluble *N*-ethylmaleimide sensitive factor attachment protein receptor (SNARE) proteins (e.g., Syntaxin-17 (STX17), Synaptosomal-associated protein 29 (SNAP29), Vesicle-associated membrane protein 7/8 (VAMP7/8)) and the Homotypic fusion and protein sorting (HOPS) complex [24,25]. Subsequently, the cargo, the autophagy receptors and inner membranes are destroyed by lysosomal hydrolase at low pH, and the debris are eventually recycled as nutrients for the cell [2]. Due to its pivotal role in the turnover of damaged, misfolded or obsolete proteins or organelles, autophagy is central to cellular homeostasis. Thus, it is no surprise that dysfunction of autophagy is associated with a wide variety of diseases, including cancer, neurodegenerative disorders and infectious diseases [5,26,27,28,29,30].

Selective autophagy has an important role in targeting invading viruses as xenophagy, a type of selective macroautophagy/autophagy that is used for eliminating invading pathogens. Thus, autophagy is currently considered an integral part of the antiviral cell-intrinsic innate immune defenses [30,31,32]. Pathogens, such as viruses, bacteria, fungi and parasites or their components are recognized by SARs and targeted for lysosomal degradation, thereby removing them from the cell [17,30,32,33,34,35]. In addition to its immediate antiviral roles, autophagy is also known to promote recognition of viruses and activation of other parts of the immune system [5]. For example, by exposing pathogen-associated molecular patterns to pattern recognition receptors (PRRs), such as Toll-like receptor 7 located in the late endosomes, autophagy may facilitate sensing viruses [36,37]^⁠^. In addition, peptides generated from viral components upon autophagic digestion are presented on antigen-presenting cells to promote antiviral adaptive immunity [38].

In summary, autophagy has emerged as a central player in innate immunity. Due to this role, autophagy has been recognized as an integral defense mechanism against viruses, including pandemic viruses, such as the severe acute respiratory syndrome virus 2 (SARS-CoV-2) and the human immunodeficiency virus 1 (HIV-1) [39,40,41].

### 1.2. Molecular Biology of HIV-1

**Figure 2:**
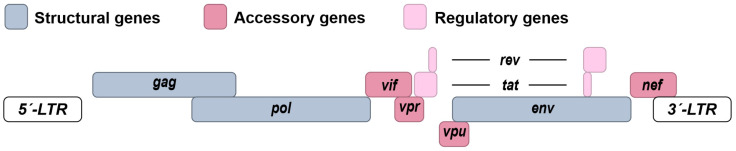
Schematic depiction of the genome of HIV-1. Group-specific antigens, *gag.* Polymerase, *pol.* Envelope, *env*. Viral infectivity factor, *vif*. Viral protein R, *vpr.* Viral protein U, *vpu.* Negative factor, *nef.* Regulator of expression of virion proteins, *rev*. Trans-activator of transcription, *tat*.

HIV-1 is the causative agent of the acquired immune deficiency syndrome (AIDS) pandemic [42,43]. HIV-1 originated from simian immunodeficiency viruses (SIVs) that were introduced to the human population by several zoonotic transmission events throughout the early 1900s [44]. Since the 1980s, HIV-1 has infected more than 75 million people worldwide with approximately 39 million individuals currently (2022) living with the infection [45]. In vivo, HIV-1 mainly infects CD4+ T cells and macrophages [46]. After an acute phase of the infection, HIV-1 becomes latent in a fraction of usually long-lived CD4+ T cells, establishing a reservoir that has the proviral DNA HIV-1 genome integrated into the host cell genome [47,48,49]. HIV-1 is one of the species of the genus Lentivirus and the family of Retroviridae. The enveloped viral particle houses two identical copies of the ~10 kB positive sense RNA genome, which is packaged and protected by the nucleocapsid protein (NP). In addition, it is associated with the viral reverse transcriptase, the integrase and the protease. The cone-shaped capsid is assembled of p24 capsid protein subunits and further protects the viral proteins and genome. The virion membrane envelope presents a surprisingly low number of about 7–11 viral glycoprotein (gp) trimers (Env) made up of gp120 and gp41 [46]. To infect the target cells, Env binds to the receptor of HIV-1, CD4, and the co-receptors CC-chemokine receptor 5 (CCR5) or CXC-chemokine receptor 4 (CXCR4), defining the tropism of HIV-1 for CD4+ T-cells and macrophages. After Env-mediated fusion between the viral and cellular membranes, the cone-shaped capsid is released into the cytoplasm. Within the cone, reverse transcription of the viral single-stranded RNA genome into linear double-stranded DNA takes place and the whole structure is imported into the nucleus via nuclear pores and disassembled. Together with cellular co-factors, the viral integrase and the proviral DNA form the pre-integration complex, and the proviral DNA is eventually integrated into the host genome, preferably in chromosomal regions that are actively transcribed [50,51]. After integration, the host-encoded machinery mediates transcription of the HIV-1 mRNAs and their subsequent translation into the viral proteins. The integrated provirus is flanked at each side by Long Terminal Repeats (LTR) serving as regulatory elements and promoters and codes for three larger polyproteins (Group-specific antigens, Gag; Polymerase, Pol; Envelope, Env), two regulatory proteins (Tans-activator of transcription, Tat and Regulator of expression of virion proteins, Rev) and four accessory proteins (Viral infectivity factor, Vif; Viral protein R, Vpr; Viral protein U, Vpu and Negative factor, Nef) (Figure 2). The Gag and Gag-Pol polyproteins are encoded by the full-length HIV-1 RNA, whereas Env as well as the regulatory and accessory proteins result from the translation of various subgenomic viral mRNAs.

The main function of the accessory proteins is to manipulate the cellular environment to favor HIV-1 replication and promote immune evasion [52]. For example, Vif promotes the degradation of the host restriction factor Apolipoprotein B mRNA editing enzyme catalytic polypeptide-like 3G (APOBEC3G); Vpu reduces Nuclear factor kappa-light-chain-enhancer of activated B cells (NF-κB) signaling, modulates DNA repair and facilitates viral release from infected cells by downregulating the restriction factor Tetherin; Vpr promotes infection of macrophages and manipulates the cell cycle; and Nef ensures T cell activation and downregulates CD4 surface expression [53,54,55,56,57,58,59,60,61]. The regulatory protein Tat promotes the expression of the viral genes and genome, and Rev facilitates the nuclear export of intron-containing viral mRNAs, including the full-length RNA genome. The structural polyproteins Gag, Pol and Env, as well as the viral genome, assemble at the plasma membrane, and immature HIV-1 virions are released. The polyproteins are autocatalytically processed after budding of the virion, and the particles mature into fully infectious HIV-1 (For a more comprehensive overview see [62,63]).

Current antiretroviral therapy (ART) has transformed HIV-1 infection from a death sentence into a manageable chronic condition. However, the latent HIV-1 reservoirs are not eliminated by current ART regimens [64]. Thus, life-long treatment is still necessary. Due to the high mutational variability of HIV-1, drug resistant strains of HIV-1 may readily emerge [65]. Thus, combinations of drugs targeting various steps of the viral replication cycle need to be used in combination [66]. Of note, using drugs targeting cellular virus dependency factors would lower the possibility of drug-resistant strains emerging [67]. However, as seen for maraviroc, which targets the host CCR5 co-receptor, resistance may still evolve in rare cases [68].

Several approaches that target the latent reservoir have been proposed. For example, the so called “shock/kick and kill” strategy relies on latency reversing agents to push HIV-1 out of hiding. Reactivated HIV-1 is then targeted for elimination, e.g., by immune mechanisms [69,70,71,72]. Alternatively, a “block and lock” approach has been proposed [70,73] that strives to permanently silence the integrated provirus locking it in the latent reservoir. However, both strategies face obstacles such as the efficiency of latency reversal or “locking” agents, treatment-induced side-effects and the need to eliminate virus-producing cells and are thus still at early stages of development [70,72]. Of note, the “block and lock” approach would still require life-long treatment.

To inspire novel curative treatments and complement ART but also to understand the pathology and life cycle of HIV-1 better, it is crucial to understand its interplay with antiviral innate immunity, such as autophagy.

## 2. The Interplay between Autophagy and HIV-1

**Figure 3:**
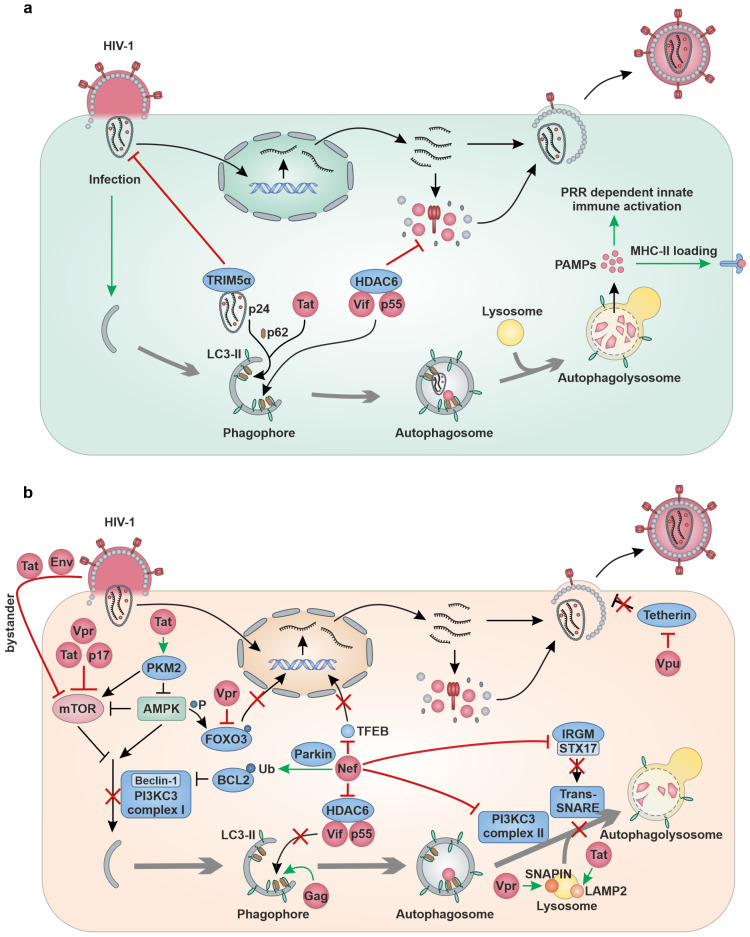
The interplay between autophagy and HIV-1 proteins. (**a**), Autophagy conveys the degradation of virions and viral proteins. For example, TRIM5α targets p24, Tat is degraded via p62 and HDAC6 targets the HIV-1 proteins Vif and p55 for autophagic clearance. Degradation of virions/viral components by autophagy provides PAMPs triggering PRR-dependent innate immune activation. Viral antigens are processed via autophagy and loaded on MHC-II molecules. (**b**), HIV-1 proteins such as Vpr, Tat and p17 trigger autophagy in infected cells. Env as well as Tat modulates autophagy in bystander cells. Tat induces autophagy via mediator PKM2-mTOR-AMPK. Vpr blocks FOXO3-mediated transcription of autophagy genes. Nef inhibits the pro-autophagic TFEB, promotes activation of BCL2, targets HDAC6 for degradation, inactivates the PI3KC3 complex II and interferes with STX17-mediated fusion of autophagosomes with lysosomes. Gag associates with LC3-II to assist its processing. Vpr triggers the degradation of SNAPIN. Tat inhibits autophagic maturation by interacting with LAMP2. Vpu mediates Tetherin restriction and promotes HIV-1 budding. Green arrows indicate positive stimulation; red arrows indicate negative regulation; Red crosses indicate inhibition.

As part of the cell-intrinsic antiviral defense mechanism, autophagy is induced upon HIV-1 infection [74,75]. Viral proteins such as Tat [76,77,78,79], Vpr [80] and p17 [81] were reported to trigger autophagy (Table 1, Figure 3a). Viral particles as well as viral proteins such as Vif [82,83], Gag [83,84] and Tat [85,86] are targeted by autophagy for lysosomal degradation.

For instance, the host factor Histone deacetylase 6 (HDAC6) forms a complex with APOBEC3G and promotes the autophagic-mediated clearance of Vif by binding Vif through its C-terminal Binder of the ubiquitin zinc finger (BUZ) domain [82,83]. Moreover, HDAC6 was shown to promote autophagic degradation of HIV-1 polyprotein p55/Gag [83,84]. The Transactive response DNA binding protein 43 kDa (TDP-43) was recently reported to stabilize HDAC6 expression and thereby support the HDAC6-mediated autophagic degradation of Vif and Gag [84]. Another restriction factor that functions as a selective autophagy receptor is the E3-ubiquitin ligase TRIM5α. Binding of TRIM5α to HIV-1 capsid protein Gag p24 induces autophagy, facilitating the recruitment of major components of the autophagic machinery such as ULK-1, Beclin-1, LC3 and p62. Eventually, this leads to autophagic degradation of the HIV-1 capsid [20]. Studies by Sagnier et al. indicate that autophagy targets HIV-1 Tat for lysosomal degradation by ubiquitin-independent interaction with the autophagy receptor p62 in CD4+ T cells [85]. However, more recently, it was also proposed that p62 binds and targets Tat marked with K63-polyubiquitination via the ubiquitin interaction domain for lysosomal degradation [86]. Besides the selective degradation of viral components from the cytosol and the resulting restriction of HIV-1 virion production, autophagy promotes further immune responses. Innate immune responses are promoted by the exposure of pathogen-associated molecular patterns (PAMPs) during autophagy. In addition, adaptive immune responses benefit from the processing of viral antigens by autophagy and presentation on Major histocompatibility complex class II (MHC-II) molecules [87,88,89] (Figure 3a). However, it is not known yet whether autophagy promotes PRR-mediated recognition of HIV-1 or whether HIV-1 derived peptides are presented in an autophagy-dependent manner on antigen presenting cells.

Increasing evidence indicates a complex role of autophagy in HIV-1 infection, suggesting modulation and manipulation of autophagy by HIV-1 on multiple levels. Depending on the cell type and the phase of infection, autophagy was proposed to exert a pro- or antiviral impact on viral spread [30,90]. Almost all HIV-1 proteins have been reported to impact autophagy (Table 1, Figure 3b). However, Nef has emerged as the major negative regulator/antagonist of autophagy. Nef is a multifunctional protein that counteracts autophagy by inhibiting early as well as late steps of autophagy in several ways. For instance, Nef recruits the E3 ubiquitin ligase Parkin (PRKN) to increase monoubiquitination of B-cell lymphoma 2 (BCL2) in CD4+ T cells. In its post-transcriptionally modified form, BCL2 strongly associates with Beclin-1 and thus inhibits the PI3KC3 complex I, which prevents autophagy initiation [91,92]. Moreover, Nef was shown to directly associate with Beclin-1 and promote mTOR activation and sequestration of pro-autophagic Transcription factor EB (TFEB) in the cytosol of macrophages [93]. Further studies by Chang et al. showed, that Nef additionally suppresses the autophagic maturation process in CD4+ T cells by inhibiting PI3KC3 complex II [94]. Structural studies revealed that Nef mimics the class III PtdIns3K complex II-binding domain (PIKBD) of Rubicon (RUBCN), an inhibitor of Beclin-1 [94]. Besides PI3KC3 complex II, the fusion of autophagosomes and lysosomes requires a complex of SNARE proteins consisting of STX17, SNAP29 and VAMP7 or VAMP8 [95]. The Immunity-related GTPase family M (IRGM) protein contributes to the assembly of SNAREs by recruiting STX17 [96]. However, it has previously been reported that Nef interacts with IRGM, leading to increased autophagosome levels [97]. In addition, Kumar and colleagues suggested that Nef interferes with STX17-IRGM interaction in macrophages, indicating an additional way by which Nef counteracts autophagic maturation [96]. Another essential function of Nef is the targeting of the host restriction factor HDAC6 for degradation, thereby protecting p55Gag and Vif from HDAC6-induced autophagic clearance [83].

Besides Nef, the other accessory proteins of HIV-1 (Vpr, Vif, Vpu) were also reported to modulate autophagy. For instance, after HIV-1 entry, virion-associated Vpr triggers the degradation of the transcription factor Forkhead box protein O3a (FOXO3a) via the ubiquitin-proteasome pathway resulting in decreased transcription of essential autophagy proteins such as LC3 and Beclin-1 and the BCL2-interacting protein 3 (BNIP3) in CD4+ T cells [98]. Phosphorylation of FOXO3 by AMPK was reported to promote nuclear translocation of FOXO3 and lead to the upregulation of those autophagy-involved genes [98,99]. In addition, Vpr also hinders the late stage of autophagy by triggering the depletion of the Synaptosome associated protein (SNAP)-associated protein (SNAPIN), a regulator of lysosomal acidification [100]. Vpr increases the pH in lysosomes and prevents autophagy-mediated degradation in neurons [101]. By targeting autophagosome formation, Vif associates with LC3 in CD4+ T cells independently of the presence of APOBEC3G and inhibits autophagy [102]. Little is known about the impact of Vpu on the autophagy process. Recently, it was proposed that the selective interaction of Vpu with LC3C in concert with ATG5 and Beclin-1 mediates the removal of the restriction factor Tetherin from budding sites [103]. The expression of the putative HIV-1 antisense protein (ASP) was reported to stimulate autophagy in monocytes and other cell lines. It was suggested that the cysteine-rich amino region of ASP mediates its multimer formation and subsequent autophagy induction [104,105].

Of note, even the regulatory (Tat and Rev) as well as the structural proteins of HIV-1 (Gag, Pol and Env) modulate autophagy. As one of the early expressed proteins after HIV-1 infection, Tat blocks Interferon (IFN)-γ-induced autophagy in macrophages by inhibiting the phosphorylation of Signal transducer and activator of transcription-1 (STAT1), resulting in decreased IFN-γ-induced expression of the autophagy gene LC3 and decreased autophagosome levels [106]. Tat was also found to colocalize with autophagosome and lysosome markers. This led to increased autophagosome but decreased LC3-II and p62 levels in neurons indicating enhanced autophagic degradation. The interaction of Tat with Lysosome-associated membrane protein (LAMP2) was proposed to enhance autophagosome and lysosome fusion to alter autophagic degradation [107]. Studies in TZM-bl cells showed that Tat can further inhibit autophagy through activation of mTOR and suppressing AMPK via the upregulation of the mediator Pyruvate kinase M2 (PKM2) [108]. Notably, Tat can be secreted by infected cells [109], pass the blood-brain barrier [110] and enter non-permissive cells like neurons via endocytosis [111]. The exposure of Tat to rodent neurons or microglial cells inhibited autophagic degradation in those cells leading to neuronal cell death and activation of microglial cells [112,113]. Thus, the impact of Tat on autophagy in cells of the central nervous system may contribute to the development of HIV-1 associated neurocognitive disorders (HAND), which is a significant clinical problem despite administration of combination ART [114]. Autophagy initiation was reported to promote optimal Gag processing [115]. Along these lines, Gag-derived proteins were found to colocalize and interact with the autophagy marker LC3 in macrophages [115]. Of note, Env was proposed to trigger autophagy in bystander macrophages and accumulation of Beclin-1 in bystander CD4+ T lymphocytes [116]. Env bystander autophagy was shown to be dependent on CXCR4 and speculated to contribute to T cell death, a hallmark of HIV-1 pathogenesis [116]. Finally, Env was proposed to inhibit autophagy in infected dendritic cells by activation of mTOR [117].

**Table 1:** Interplay between HIV-1 proteins and autophagy.

Viral Protein	Targeted by Autophagy	Impact on Autophagy
Gag	HDAC6-mediated autophagic degradation of p55 in transfected HEK293T [83,84]. Degradation of p24 by autophagy in a TRIM5α-dependent manner in rhesus CD4+ T cells and Langerhans cells [20,118].	Association with LC3 and promotion of Gag processing via autophagy in macrophages [115]. Autophagy activation by p17 in lymph node-derived lymphatic endothelial cells [81].
Vif	HDAC6-mediated autophagic degradation in transfected HEK293T [82,84].	Inhibition of autophagy by interaction with LC3 in CD4+ T cells [102].
Vpr		Autophagy activation in transfected macrophages [80]. Inhibition of autophagic maturation by inducing SNAPIN degradation in neurons [101]. Inhibition of autophagic nucleation by inducing FOXO3a degradation in CD4+ T cells [80,98].
Tat	Degradation in a p62-dependent manner in CD4+ T cells, potentially ubiquitin dependent [85,86].	Activation of autophagy in astrocytes, glial cells, microglial cells and endothelial cells [76,77,78,79]. Modulation of autophagic maturation by association with LAMP2 in neurons [107]. Inhibition of IFN-γ mediated autophagy in macrophages [106]. Inhibition of autophagy by activating mTOR in TZM-bl [108]. Inhibition of late steps of autophagy and triggering cell death in rat neurons [112]. Inhibition of mitophagy in mouse microglia [113]. Activation of autophagy in bystander cells via the AKT-STAT3 axis [119].
Vpu		Exploits components of the autophagic machinery to mediate Tetherin restriction [103].
Env		Activates autophagy in non-infected bystander CD4+ T cells [85,116,120]. Activation of mTOR in infected dendritic cells [117].
Nef		Inhibition of autophagy nucleation by inducing ubiquitination of BCL2 in CD4+ T cells [91,92]. Inhibition of autophagic nucleation by inducing sequestration of TFEB in macrophages [93]. Inhibition of autophagic maturation by mimicking the class III PtdIns3K complex II-binding domain (PIKBD) of RUBCN, an inhibitor of Beclin-1 in CD4+ T cells [94]. Autophagic degradation of HDAC6 in Nef transfected HEK293T [83]. Inhibition of autophagic maturation by interference with STX17-IRGM interaction in macrophages [97].
ASP		Autophagy induction in infected cells [104,105].

## 3. Cell-Type-Specific Effects

**Figure 4:**
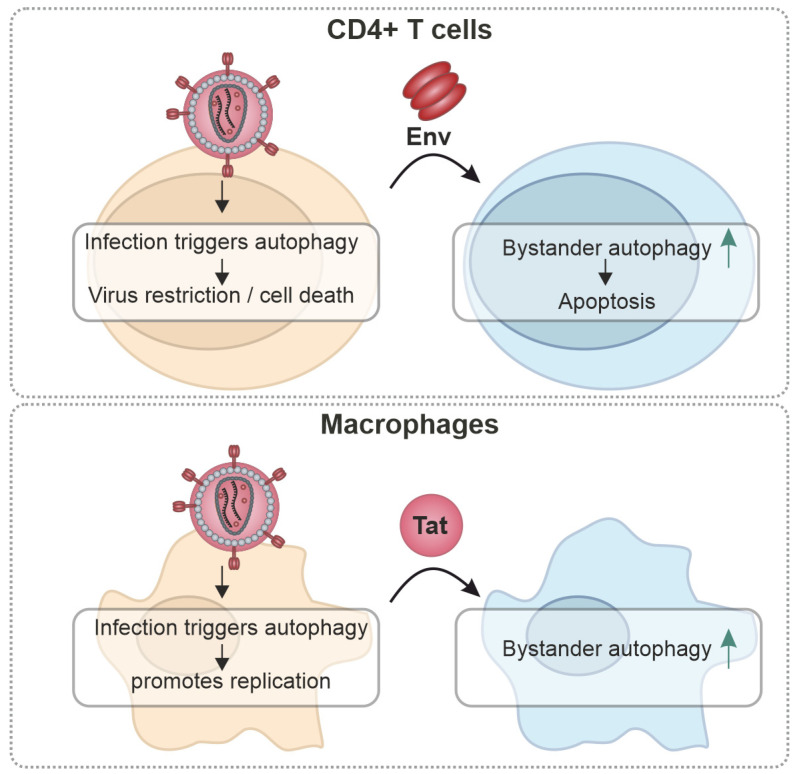
The proposed cell-type-specific role of autophagy in HIV-1 infection. In CD4+ T cells, infection initiates a mainly antiviral autophagy response, which contributes to cell death of the infected cells. Released Env triggers autophagy in bystander CD4+ T cells, causing apoptosis. In infected macrophages, autophagy is activated and promotes HIV-1 replication. Secreted Tat modulates autophagy in bystander macrophages.

The main target cells of HIV-1 are CD4+ T cells and (to a lesser extent) macrophages [121]. In both of these cell types, autophagy—besides its role in the innate defenses—plays essential homeostatic functions. For example, autophagic flux is required for T-cell activation and differentiation and macrophage differentiation (Figure 4) [122,123,124,125,126,127]. In infected CD4+ T cells, autophagy has mainly antiviral roles, and activation of autophagy leads to decreased viral replication, e.g., selectively degrading Tat (Figure 4) [85,115,120]. Similarly, inhibition of autophagy in dendritic cells promotes HIV-1 replication, enhanced HIV-1 transfer to CD4+ T cells and decreased MHC-II mediated HIV-1 antigen presentation to CD4+ T cells [117]. In contrast, in infected glial cells and macrophages, autophagy seems to be induced by HIV-1 to sustain cell survival [76,80] and was shown to be required for efficient HIV-1 production [115,120]. Here, Gag colocalizes with autophagosomes and it has been suggested that autophagy is required for optimal processing [115,120].

Importantly, HIV-1 also modulates autophagy in a cell-type-specific manner not only in the infected but also in bystander cells. Bystander autophagy induction by Env was suggested to promote apoptosis in CD4+ T cells and may contribute to the loss of (bystander) CD4+ T cells [85,116,120]. Of note, it was reported that Env-mediated bystander autophagy is selective for CD4+ T cells [116,120,128,129]. In macrophages, bystander autophagy was reported to be activated by Tat through AKT-STAT3 signaling [119]. Interestingly, despite activating bystander autophagy, macrophages do not appear to be significantly depleted during the course of an HIV-1 infection, unlike CD4+ T cells [130].

Altogether, emerging evidence suggests that autophagy may affect HIV-1 differentially in both types of its main physiological target cells [90,120,130]. Whereas in T cells, autophagy is predominantly antiviral, in macrophages autophagy seems to have a dual role: it is required for optimal virus production/infectivity but degradation of the particle by autophagic turnover needs to be avoided [90]. However, more research is needed to dissect the cell-type and tissue-specific interplay of autophagy and HIV-1.

## 4. Autophagy Modulation as an Antiviral Approach

Considering the role of autophagy as part of the innate immune defenses, but also as viral dependency mechanism, both therapeutic activation and inhibition of autophagy may have an antiviral impact. Thus, targeting autophagy has been suggested to act as a double-edged sword against viruses [30]. Therapeutic modulation of autophagy can be achieved by a variety of compounds [131]. For example, the naturally occurring rapamycin, which was isolated from *Streptomyces hygroscopicus*, inhibits mTORC1 and thus induces autophagic flux [132,133]. Artificial analogs of rapamycin, such as temsirolimus (CCI-779), everolimus (RAD-001) and deforolimus (AP-23573), use the same targeting strategy [134]. To achieve a more stringent inhibition of the mTOR complex, i.e., targeting of both the mTORC1 and mTORC2 subunit, ATP-competitive mTOR inhibitors (e.g., PP242, AZD8055, WYE132) and the dual PI3K-mTOR inhibitor NVP-BEZ235 were developed [135]. Trehalose, a naturally occurring sugar, was shown to induce autophagy in an mTOR-dependent and mTOR-independent fashion by TFEB activation [136]. Metformin, an antidiabetic drug, targets and inhibits AMPK upstream of the mTOR complex [137]. While compounds activating autophagy are mainly limited to the AMPK-mTOR axis, autophagy inhibition can be achieved by a wider variety of drugs. Early stages of autophagy are inhibited by 3-methyadenine (3-MA), Wortmannin, LY294002 and PIK-III, while late stages are suppressed by chloroquine (CQ), hydroxychloroquine (HCQ), or bafilomycin A1 that prevent fusion of autophagosomes with the lysosomes. 3-MA, wortmannin, LY294002 and PIK-III all target the class III PI3K (Vps34), whereas bafilomycin A1 and chloroquine prevent the acidification of lysosomal compartment and/or formation of autophagolysosomes [138,139]. In addition, the ‘specific and potent autophagy inhibitor 1′ (Spautin-1) binds to ubiquitin-specific peptidases (USP) 10 and 13 and promotes the ubiquitin-mediated degradation of Beclin-1 [140].

Therapeutic modulation of autophagy has been extensively explored in cancer therapy [29]. However, activation of autophagic flux, e.g., by mTOR inhibitors as a monotherapy, has turned out to be of limited efficacy. Combinatorial therapy with cytotoxic chemotherapy or radiation therapy was shown to have a promising impact in vitro. Inhibition of autophagy may promote cell death of highly proliferating tissue, thus enhancing tumor cell death and complementing existing cytotoxic chemotherapy. Currently, several phase I/II trials evaluate the combination of HCQ with cytotoxic drugs in patients with brain, lung, breast, colorectal, pancreas, kidney and prostate cancers [29,141]. While already approved in cancer therapy, there are currently no antiviral therapies based on autophagy modulation.

In vitro studies showed that despite inhibition of autophagy by several viral proteins, pharmacological activation of autophagy can overcome these antagonists leading to the autophagic-degradation of HIV-1 capsid proteins and a decrease in virion release through an ATG5- and autophagy-dependent mechanism [142,143]. For example, HIV-1 is restricted by rapamycin-induced autophagy in ex vivo cultures [144]. Other mTOR inhibitors like vorinostat, panobinostat, givinostat and romidepsin and the non-histone chromatin modulating Bromodomain and extra terminal (BET) inhibitor JQ1, dactolisib (NVP-BEZ235), and SF2523 were reported to decrease both intracellular and extracellular HIV-1 capsid protein in an autophagy-dependent manner [142,145,146]. Induction of autophagy by an artificial Tat-Beclin-1 derived peptide restricts HIV-1 replication in an autophagy-dependent manner [143]. The autophagy inducer trehalose was reported to induce degradation of intracellular HIV-1 capsid proteins and an autophagy-dependent reduction in HIV-1 release [147]. In addition, Second mitochondria-derived activator of caspase (SMAC) mimetics were shown to promote autophagy-dependent apoptosis of HIV-1-infected macrophages [148]. Recently, miRAB40, an autophagy-inducing miRNA upregulated by Interleukin-27 was suggested to restrict HIV-1 via regulation of autophagy [149]. Similarly a non-silencing miRNA directed against HIV-1 Gag was suggested to induce autophagic degradation of the virion [150]. Specific autophagy-related factors involved in HIV-1 replication can also be therapeutically targeted. For example, it was suggested that engineered HIV-1 restricting rhesus monkey TRIM5α could be introduced in gene therapeutic approaches due to their ability to directly target components of HIV-1 for autophagic degradation via precision autophagy [151,152]. Finally, activation of autophagy is thought to not only limit the replication of HIV-1 but also prevent the initial infection. It was reported that autophagy-enhancing drugs limit mucosal HIV-1 acquisition and suppress viral replication ex vivo [153]. Thus, autophagy activating compounds such as mTOR inhibitors could be used as agents to complement ART. However, the in vitro efficiency as well as the specificity of currently available drugs to modulate autophagy does not (yet) match currently available HIV-1 therapeutics.

However, the biggest challenges in HIV-1 therapy is the elimination of the latent reservoir to achieve a cure. As autophagy does not impact transcriptionally latent HIV-1, all strategies explored so far only target actively replicating or incoming HIV-1. However, it has been suggested that autophagy modulation may complement strategies to target the reservoir, thereby enhancing their efficiency [142]. A lipid-coated hybrid poly(lactic-co-glycolic acid) (PLGA) nanoparticle loaded with the Tat-Beclin-1 peptide was reported to preferentially induce cell death of latently infected CD4+ T cells via autophagic-cell death induction (autosis) [143,154,155]. Similarly, autosis of latently infected cells was induced by nanoparticle-encapsulated v-FLIP-α2 peptide [155,156]. Modulation of autophagy may also enhance ‘shock and kill’ approaches, where latently infected reservoirs are first reactivated and then eliminated. It was shown that in combination with agents that promote latency reversal, selective killing of reactivated T cells can be achieved by autophagy inhibition via chloroquine or SAR405 [157,158].

In summary, various strategies that activate autophagy have been shown to restrict the replication of HIV-1, and autophagy inhibition may complement current efforts that target the latent reservoir.

## 5. Concluding Remarks

As part of the innate immune defenses, autophagy contributes to the restriction of HIV-1 [41,130]. However, similar to other successful human pathogens, HIV-1 evolved strategies to circumvent and even exploit autophagy. Nef can be considered the main autophagy antagonist encoded by HIV-1, inhibiting both initiation and autophagy turnover [91,94]. Notably, Nef targets two seemingly similar complexes (PIKC3-Complex I and II), which also share components. However, two different mechanisms are employed: Activity of PI3K-CI is inhibited by activating the autophagy inhibitor Bcl-2 [91,92]. To prevent activation of the PI3KC3-CII, Nef uses molecular mimicry, adopting a structure similar to RUBCN, an inhibitor of Beclin-1 [94]. However, Nef is by far not the only protein of HIV-1 that manipulates autophagy. All accessory proteins and most structural and regulatory proteins were reported to impact autophagy using various mechanisms (Table 1). Thus, multilevel control of autophagy seems of high importance for HIV-1.

While the impact of HIV-1 on autophagy may seem ambivalent and complex, it involves two basic strategies: (I) Preventing autophagic turnover to avoid lysosomal degradation of virions or viral components and (II) exploitation of parts of the autophagic machinery responsible for membrane rearrangements and trafficking functions to promote replication. For example, Nef inhibits autophagy to reduce the degradation of viral protein products [82,94,96]. In contrast, Vpu utilizes the autophagic machinery to counteract Tetherin [103], and in macrophages, autophagy is exploited for Gag processing [115]. This approach—inhibiting the antiviral function and hijacking useful parts—is also reflected in the seemingly ambiguous roles of some HIV-1 proteins in autophagy. For example, the matrix protein p17 was reported to induce autophagy but also suppress autophagy [81]. Of note, dual roles of viral proteins in autophagy may also be partially explained by cell-type-specific effects. However, this requires future research.

Considering the anti- and proviral roles of autophagy, it has been proposed that both activation and inhibition of autophagy may restrict HIV-1 [30]. Despite encoding multiple inhibitors of autophagy, induction of autophagy overwhelms viral antagonism of autophagy [145,151]. The virus may be most vulnerable towards autophagy induction especially during the early steps of the infection, before the de novo expression of accessory proteins that modulate autophagy. In contrast, broad inhibition of autophagy was reported to increase the infectivity of HIV-1 [115]. Thus, despite occasionally seemingly proviral roles of autophagy, inhibition of autophagic flux is per se not antiviral. However, components of the autophagic machinery that are exploited by HIV-1, i.e., host-dependency factors, could identify so far unexplored targets for therapy [152]. It has been proposed that modulation of autophagy may also aid cure strategies [64,142,158]. Indeed, host-directed autophagy-inhibiting drugs were shown to increase autosis (autophagic cell death) [154]. In combination with latency reversal agents, this may improve viral reservoir targeting as part of the “shock and kill” approach [142]. However, curative approaches involving autophagy still face major obstacles as the drugs would need to reach potentially isolated latent reservoirs (e.g., the brain), the efficiency and specificity of latency-inducing agents and selectively autosis-inducing treatments need to be substantially improved as well.

Of note, modulation of autophagy may augment and complement existing ART regimes. However, it needs to be noted that current drugs to activate autophagy are less efficient against HIV-1 than currently approved drugs, judging from in vitro data. Furthermore, activation of autophagy by the currently available compounds is not selective for infected cells. In addition to a direct antiviral impact, activation of autophagy may promote the formation and/or maintenance of B and T cells [123,124,125,159] enhancing humoral and cellular anti-HIV adaptive immune responses. In addition, autophagy has anti-inflammatory properties [5] and may serve as an auxiliary therapy to combat inflammation that is often a complication of ART [160]. There may be a caveat though. As HIV-1 mainly infects and perturbs signaling in immune cells, additional exogenous manipulation of autophagy may have an unexpected impact on organismal cytokine and inflammation homeostasis.

It is likely that HIV-1 targets non-canonical autophagy as well, such as LC3-associated phagocytosis (LAP) [4,127]. For example, Vpu recruits LC3C at the budding site to remove Tetherin by a non-canonical autophagy reminiscent of LAP [103,161]. However, future research is needed to understand and clearly dissect the roles of canonical and non-canonical autophagy in HIV-1 replication.

Manipulation of autophagy as a host defense mechanism against HIV-1 may extend beyond the infected cells [116,119]. Env-mediated autophagy induction in bystander CD4+ T cells was suggested to contribute to bystander cell death, despite autophagy being a pro-survival mechanism [116,120]. Nevertheless, it is tempting to speculate that HIV-1-mediated bystander autophagy manipulation is part of the efforts to create a viral replication niche, i.e., favorable conditions for local replication and spread. However, the experimental evidence for this, as well as broader implications, are currently unexplored.

Are autophagy and HIV-1 friends or foes? Certainly, a bit of both, but rather rivals than lovers caught in an evolutionary dance. Future research is clearly needed to unravel the interplay between autophagy and HIV-1 to answer the remaining questions: Can the differential impact of autophagy in macrophages and T-cells be dissected? What are the precise molecular mechanisms and impact of bystander autophagy? Can autophagy modulation be part of curative strategies or improved to augment ART? Exploring these questions will certainly improve our understanding of the molecular pathogenesis of HIV-1 and may inspire novel therapeutic approaches based on autophagy modulation.

## Data Availability

No data was created for this review.
